# Possible mechanisms for sensorineural hearing loss and deafness in patients with propionic acidemia

**DOI:** 10.1186/s13023-017-0585-5

**Published:** 2017-02-13

**Authors:** S. C. Grünert, I. Bodi, K. E. Odening

**Affiliations:** 1 Center of Pediatrics and Adolescent Medicine, Medical Center – University of Freiburg, Faculty of Medicine, University of Freiburg, Mathildenstr. 1, 79106 Freiburg, Germany; 2 Department of Cardiology and Angiology I, University Heart Center Freiburg, Freiburg, Germany

**Keywords:** Propionic acidemia, Propionic aciduria, Hearing loss, Long QT syndrome, Sensorineural deafness, KvLQT1, KCNE1, Voltage-gated potassium channel, Inner ear

## Abstract

Propionic acidemia is an inborn error of metabolism caused by deficiency of the mitochondrial enzyme propionyl-CoA carboxylase. Sensorineural deafness and severe hearing loss have been described as long-term complications of this disease, however, the mechanism has not yet been elucidated. We have recently shown by patch clamping experiments and Western blots that acute and chronic effects of accumulating metabolites such as propionic acid, propionylcarnitine and methylcitrate on the KvLQT1/KCNE1 channel complex cause long QT syndrome in patients with propionic acidemia by inhibition of K^+^ flow via this channel. The same KvLQT1/KCNE1 channel complex is expressed in the inner ear and essential for luminal potassium secretion into the endolymphatic space. A disruption of this K^+^ flow results in sensorineural hearing loss or deafness. It can be assumed that acute and chronic effects of accumulating metabolites on the KvLQT1/KCNE1 channel protein may similarly cause the hearing impairment of patients with propionic acidemia.

Propionic acidemia (PA) is an inborn error of metabolism caused by deficiency of the mitochondrial enzyme propionyl-CoA carboxylase. This enzyme converts propionyl-CoA to D-methylmalonyl-CoA and is involved in the metabolism of branched-chain amino acids, odd-numbered fatty acids, cholesterol side chains, thymine, and uracil [1]. Patients with PA are prone to acute life-threatening metabolic decompensations with severe metabolic acidosis and hyperammonemia, and most affected children already present with symptoms during the neonatal period. With progression of the disease, long-term complications involving the neurological, cardiological, hematological, immunological and gastrointestinal system are common [2]. One of the main cardiological problems is the development of an acquired long QT syndrome (LQTS), which can be found in up to 70% of patients beyond childhood and puts patients at risk for potentially lethal ventricular arrhythmia [2, 3]. Among the neurological complications, sensorineural hearing impairment has been reported in several patients with PA [2, 4, 5]. In a study with 55 PA patients from Germany, Switzerland and Austria, hearing loss was apparent in 13% of the patients [2]. Similarly, Brosch et al. described 4 patients with PA and sensorineural deafness or severe hearing loss [5]. Mutation analyses of the *PCCA* and *PCCB* genes, encoding the two subunits of the propionyl-CoA carboxylase, have been performed in the families of these patients to elucidate whether mutations primarily responsible for PA could also be the underlying cause for a genetic form of deafness. Brosch et al. concluded that no connection can be assumed between the mutations found in the patients and the severe sensorineural hearing loss. So far, the mechanism of hearing impairment in PA has not yet been elucidated. However, our studies on the effects of metabolites, that typically accumulate in body fluids of patients with PA, on the KvLQT1/KCNE1 channel give new insights on the possible mechanism of hearing loss associated with this disorder.

The same combination of LQTS and sensorineural deafness as seen in PA patients is also found in Jervell and Lange-Nielsen syndrome. This genetic disorder with an autosomal recessive inheritance is characterized by congenital LQTS with syncopal attacks due to ventricular arrhythmias and congenital profound bilateral sensorineural hearing loss [6]. It is caused by mutations in either the *KCNQ1* or *KCNE1* genes, encoding the alpha and beta subunits (KvLQT1 and KCNE1/minK) of a potassium channel carrying the so-called delayed rectifier potassium current I_Ks_ [7]. About 90% of cases of Jervell and Lange-Nielsen syndrome are caused by mutations in the *KCNQ1* gene [8–10]. The KvLQT1/KCNE1 voltage-gated potassium channel is expressed not only in cardiomyocytes but also in cells of the stria vascularis of the inner ear [11, 12]. In the heart, this channel is essential for cardiac repolarization, and mutations in either the *KCNQ1* or *KCNE1* gene lead to a reduction of the I_Ks_ current and therefore prolongation of the action potential [13, 14]. In the inner ear, this channel enables luminal secretion of K^+^ into the endolymphatic space [15, 16]. A disruption of this K^+^ flow results in sensorineural hearing loss or deafness. We therefore hypothesize that sensorineural hearing loss and long QT syndrome seen in propionic acidemia share a common pathogenic mechanism.

We have recently shown that the acquired LQTS observed in patients with PA is due to acute and chronic effects of accumulating metabolites on the KvLQT1/KCNE1 channel and its function. Namely, propionic acid, propionylcarnitine and methylcitrate, the key metabolites accumulating in PA, have acute blocking effects on this potassium channel and thereby reduce I_Ks_ current and prolong action potential duration (Fig. 1) [17]. Additionally, the expression of KvLQT1 is altered in the presence of these metabolites: Chronic exposure (24 h) to propionylcarnitine and methylcitrate decreases KvLQT1 expression - in line with the acute I_Ks_-blocking effects [17]. We hypothesize that the hearing impairment of patients with PA may be caused by the same mechanisms that lead to acquired LQTS (Fig. 2). In the inner ear the KvLQT1/KCNE1 channel is critical for the production and homeostasis of the endolymph, the potassium-rich fluid surrounding the neuroepithelium of the inner ear [6]: Potassium ions are absorbed from the perilymph by basal cells, further transported through gap junctions to intrastrial fluids, and then pumped into intermediate cells through the Kir4.1 potassium channel, which creates a high intracellular potassium concentration [6]. The consequential concentration gradient between intrastrial fluid and intermediate cells generates the endocochlear potential [18]. From the intrastrial fluid, potassium ions are transported across the basolateral membrane of marginal strial cells by the Na^+^/K^+^-ATPase and Na^+^/K^+^/Cl^−^ cotransporter [19–22]. Finally, K^+^ crosses the apical membrane through the KCNQ1/KCNE1 potassium channel and is secreted into the endolymph [11, 19]. The endocochlear potential is essential for audition [20] and disturbances of the endocochlear potential can lead to the immediate loss of hearing, which may be irreversible [23].

**Fig. 1:**
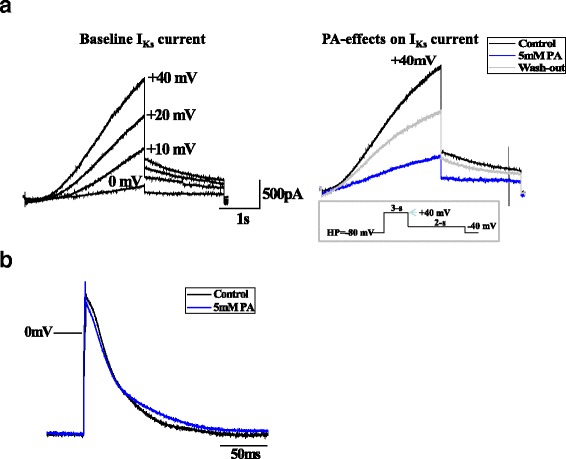
**a**. Propionic acid effects on I_Ks_ current in KCNQ1/KCNE1-CHO cells. Representative whole cell current traces at baseline, after the application of PA (5 mM, *blue*), and after brief wash-out (*grey*), demonstrating an acute reduction of I_Ks_ by 50%. Voltage clamp protocol is shown in the inset. **b**. Propionic acid effects on action potential in human iPSC-CMs. Superimposed representative AP traces at baseline and after PA (5 mM, *blue*), indicating an AP-prolongation

**Fig. 2:**
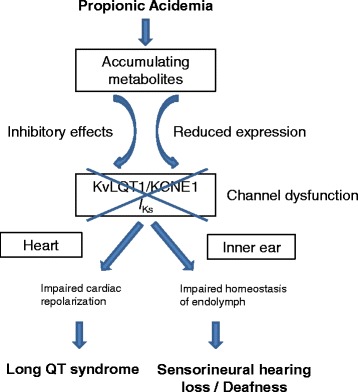
Proposed pathophysiologic mechanism of long QT syndrome and sensoneurinal hearing loss in propionic acidemia. Accumulating metabolites such as propionic acid, methylcitrate and propionylcarnitine cause dysfunction of the KvLQT1/KCNE1 channel by acute blocking effects as well as reduced expression of the channel protein

In addition to altered KCNQ1/KCNE1 channel function, histopathological changes in the inner ear - causatively linked to the altered KCNQ1/KCNE1 function - have been described in patients with Jervell and Lange-Nielsen syndrome: As early as in the 1960ies, temporal bone histology has been investigated and profound histopathologic abnormalities including the collapse of Reissner’s membrane and membranes surrounding the saccule, utricle and ampullae were found, resulting in the obliteration of the scala media and endolymphatic compartments of the vestibular end organs [6, 24, 25]. KCNQ1 knockout mice similarly exhibit histopathologic findings that are comparable to those reported in patients with Jervell and Lange-Nielsen syndrome with marked atrophy of the stria vascularis, contraction of the endolymphatic compartments, collapse and adhesion of surrounding membranes, complete degeneration of the organ of Corti and an associated degeneration of the spiral ganglion [26]. These histopathological changes underline the importance of the KvLQT1/KCNE1 channel for the structural integrity of the inner ear.

Due to the critical role of the KvLQT1/KCNE1 channel, it is well conceivable that blocking effects of circulating metabolites of propionic acid on the I_Ks_ current as well as effects on the expression of the channel subunits may have severe consequences and lead to hearing impairment or even deafness. These mechanisms are likely to be the cause of hearing impairment in patients with PA.

## Abbreviations

LQTS: Long QT syndrome
PA: Propionic acidemia
